# Comparison of the efficacy of intranasal ketamine versus intravenous ketorolac on acute non-traumatic headaches: a randomized double-blind clinical trial

**DOI:** 10.1186/s13005-021-00303-0

**Published:** 2022-01-03

**Authors:** Hooman Rafiei Sarvari, Hamidreza Baigrezaii, Mohammad Nazarianpirdosti, Amirhossein Meysami, Roya Safari-Faramani

**Affiliations:** 1 Department of Emergency Medicine, School of Medicine, Kermanshah University of Medical Sciences, Kermanshah, Iran; 2 Student Research Committee, Kermanshah University of Medical Sciences, Kermanshah, Iran; 3 Department of Epidemiology, School of Health, Kermanshah University of Medical Sciences, Kermanshah, Iran

**Keywords:** Emergency department, Headache, Intranasal, Intravenous, Ketamine, Ketorolac, Non-traumatic, Randomized clinical trial

## Abstract

**Introduction:**

Non - traumatic headaches are one of the most common causes of referral to hospital emergency. This study aimed to compare the efficacy of intranasal ketamine and intravenous ketorolac on acute non-traumatic headaches.

**Methods:**

This randomized and double-blind clinical trial was conducted in 2019. One hundred and forty samples were randomly divided into intranasal ketamine (A) and intravenous ketorolac (B). Group (A) received ketamine intranasal (0.75 mg/kg, max 75 mg), and group B received intravenous ketorolac (30 mg). Headache severity was measured on arrival, 30, 60, and 120 min after intervention with Visual Analogue Scale (VAS). The side effects were recorded an hour after the intervention.

**Result:**

The mean difference of pain intensity 30, 60, and 120 min after the intervention between the two groups was statistically significant (*p* < 0.001). In the first 30 min, significant changes were observed in the VAS levels of the two groups. These changes were significantly greater in the intranasal ketamine group (*p* < 0.001). Side effects such as fatigue, dizziness, general discomfort, nausea, increased heart rate, and hypertension were significantly higher in the ketamine group (*p* < 0.05).

**Conclusion:**

Intranasal ketamine and intravenous ketorolac both effectively reduced headaches. However, more analgesic effects of intranasal ketamine in a short time can be considered as a selective approach to reducing headaches.

**Trial registration:**

IRCT20180108038276N3, Registered 29 September 2019.

**Ethics committee reference number:**

IR.KUMS.REC.1398.068.

## Introduction

Headache is one of the most common complaints of patients referring to outpatient clinics and emergency departments [1]. About 50% of the world’s population suffers from headaches. This has led to concerns from the World Health Organization (WHO) [2]. In patients referred to the emergency department (ED), the prevalence of non - traumatic headaches is 0.5 to 5.4%, which is considered a big diagnostic challenge [3]. The treatment methods used to treat and control headaches are varied and include pharmacological and non-pharmacological treatments. Narcotics and acetaminophen are among the pharmacological treatments used [4, 5], and these may have side effects (e.g., respiratory problems, nausea, and vomiting), today, newer drugs are being used that effectively reduce pain and have fewer side effects [6]. One of the most commonly used medications to control pain in the emergency room is Ketamine. It is injected into intramuscular, intravenous, and intranasal forms [7, 8]. Ketamine, in addition to a significant reduction in pain, has also been introduced as an effective remedy for headache control [9, 10]. Ketorolac is another common drug used in an emergency for headaches management and is used in intramuscular, intravenous, or oral forms [11, 12]. Numerous studies have been performed to evaluate these drugs’ effectiveness, but so far, no recommended drug in this regard has been selected [9, 13–17].

Since so far no study has been done to compare the effect of intranasal ketamine and intravenous ketorolac on the severity of headache; and also, to provide effective and useful evidence in decision making for better and practical intervention to headache management, the present study was designed to compare the efficacy of intranasal ketamine and intravenous ketorolac on acute non-traumatic headaches.

## Materials and methods

### Study design

The present study was a randomized, double-blind clinical trial with parallel design and a 1:1 allocation ratio in the intranasal ketamine and intravenous ketorolac groups. The study was conducted based on the CONSORT guidelines.

### Sample and sampling method

The study population included all patients with non - traumatic acute headaches referred to Imam Reza Hospital based in Kermanshah, Iran. The sample size is calculated according to Meredith et al.s’ and Zitek et al.s’ studies with a confidence level of 95% and a test power of 80% [17, 18]. The number of samples required in each group was 35 subjects. To increase the reliability and ensure sufficient study power, 70 subjects were assigned to each group. Thus, 140 subjects in total were considered in the study. Among these samples, 70 were considered for the ketamine intranasal intervention group and 70 for the intravenous ketorolac intervention group. The inclusion criteria were primary (Migraine, tension, and cluster) [19], being in the 18–65 age group, with self-reported severity of 4 or greater on a Visual Analogue Scale (VAS) (0–10), and willingness to participate in the study. Exclusion criteria were weight < 45 kg or > 115 kg, vital sign abnormalities including heart rate < 50 beats/min or > 150 beats/min, systolic blood pressure < 80 or > 200 mmHg, oxygen saturation < 92%, or respiratory rate < 8 or > 30 breaths/min, patient with a history of alcohol abuse, intracranial hypertension, ischemic heart disease, human immunodeficiency virus or immunosuppression, renal diseases requiring dialysis, liver disease, poorly controlled thyroid disease, active bleeding, or current use of anticoagulants [20]. Also, patients with a functional neurological disorder (FND), headache with an impaired level of consciousness, headaches with comorbidity, pregnant and lactating women were excluded from our study.

### Measurement instrument

The study instrument included a questionnaire consisting of 3 parts. The first part was related to the demographic information (including age, sex, history of drug sensitivity, and history of headache). In the second part, the heart rate, blood pressure, fatigue, dizziness, general discomfort, and nausea were recorded one hour after the intervention. The third section was devoted to recording patients’ pain scales based on VAS before prescribing the drug, 30 min, 60 min, and 120 min after receiving the drug. Visual Analogue Scale (VAS) is the same pain ruler with a horizontal line graded from 0 to 10 [21]. VAS is the most widely used and easiest means of measuring pain in the world, whose validity and reliability, both English and Persian versions, have been reviewed and approved in previous studies [22, 23]. 0 non-pain, 1–3 mild pain, 4–6 moderate pain, 9–7 severe pain, and 10 indicate the most severe pain according to this tool [24, 25].

### Intervention

Approval was first obtained from the Ethical Review Committee of Kermanshah University of Medical Sciences (KUMS), and then sampling was conducted. After taking a history and physical examination of patients by the emergency medicine resident and evaluating them in terms of inclusion and exclusion criteria, qualified samples were entered into the study. The sampling method was simple random. After identifying the qualified patients, they were randomly assigned a specific three-digit code. The last digit on the right of the three-digit code determined the patient’s group. If this number was 0, 1, 2, 3, or 4, patients belonged to the intravenous ketorolac group, and if it was 5, 6, 7, 8, or 9, they belonged to the intranasal ketamine group. Thus, 70 people were assigned to the intranasal ketamine group, and 70 people were assigned to the intravenous ketorolac group. At first, the socio-demographic information form and the pain intensity of the patients were completed and recorded. After a practiced nurse placed the IV, ketorolac-treated patients received 30 mg IV ketorolac, and ketamine-treated patients received 0.75 mg/kg (maximum 75 mg) intranasal ketamine via a MAD Nasal™ intranasal mucosal atomization device affixed to a 10-cc syringe (Teleflex Medical Europe Ltd., Westmeath, Ireland) [20, 26]. A specific medication was prepared, and its dose was determined by the triage nurse based on the patient’s code. It was administered by the emergency physician, who was unaware of the study protocol. Subjects allocated to the intranasal ketamine arm received 1000 mL of normal saline in a bag identical to that administered to the intravenous ketorolac arm for subject blinding. Subjects in the intravenous ketorolac arm also inhaled a dose of atomized intranasal saline (0.015 mL/kg, maximum 1.5 mL) via a MAD Nasal™ intranasal mucosal atomization device affixed to a 10-cc syringe (Teleflex Medical Europe Ltd., Westmeath, Ireland). We did not blind nursing staff to the patients’ medications, though we did blind patients and investigators.

### Outcome

The VAS (from 0 cm: painless to 10 cm: the most severe pain) was used to measure the pain. On arrival, 30, 60, and 120 min after intervention, the sheet containing VAS was given to patients and asked to mark their level of pain. Also, the side effects of the drugs were recorded one hour after the intervention. The patients were asked to report if they have any side effects. These side effects included fatigue, dizziness, general discomfort, and nausea [20, 27]. In addition to these side effects, increased heart rate and blood pressure were measured and recorded [28]. The patients also were asked to inform the investigator if they have any other unpleasant sensations.

### Data analysis

Data analysis was performed with descriptive statistics (mean, percentage, and standard deviation), and analytical statistics Kolmogorov-Smirnov test, Kruskal-Wallis test, Wilcoxon signed-rank test, Mann-Whitney U test), and SPSS statistical software version 18.0 (SPSS Inc., Chicago, IL, USA). First, the normality of the data was investigated by the Kolmogorov-Smirnov test. To compare the pain severity in different time ranges, repeated measures of ANOVA, and to compare side effects between the two groups, the t-test was used. A significant level was determined < 0.05.

### Ethical considerations

Approval was obtained from the Ethical Review Committee of Kermanshah University of Medical Sciences with reference number: IR.KUMS.REC.1398.068, and also registered at the Iranian Registry of Clinical Trials on 29 September 2019, with the registration number: IRCT20180108038276N3, and URL:(https://en.irct.ir/trial/41516). Details of the study included the aim, intervention process, and confidentiality of the explicitly described information to all subjects. Subjects that were willing to participate in this study entered the study after obtaining written consent.

## Results

**Fig. 1:**
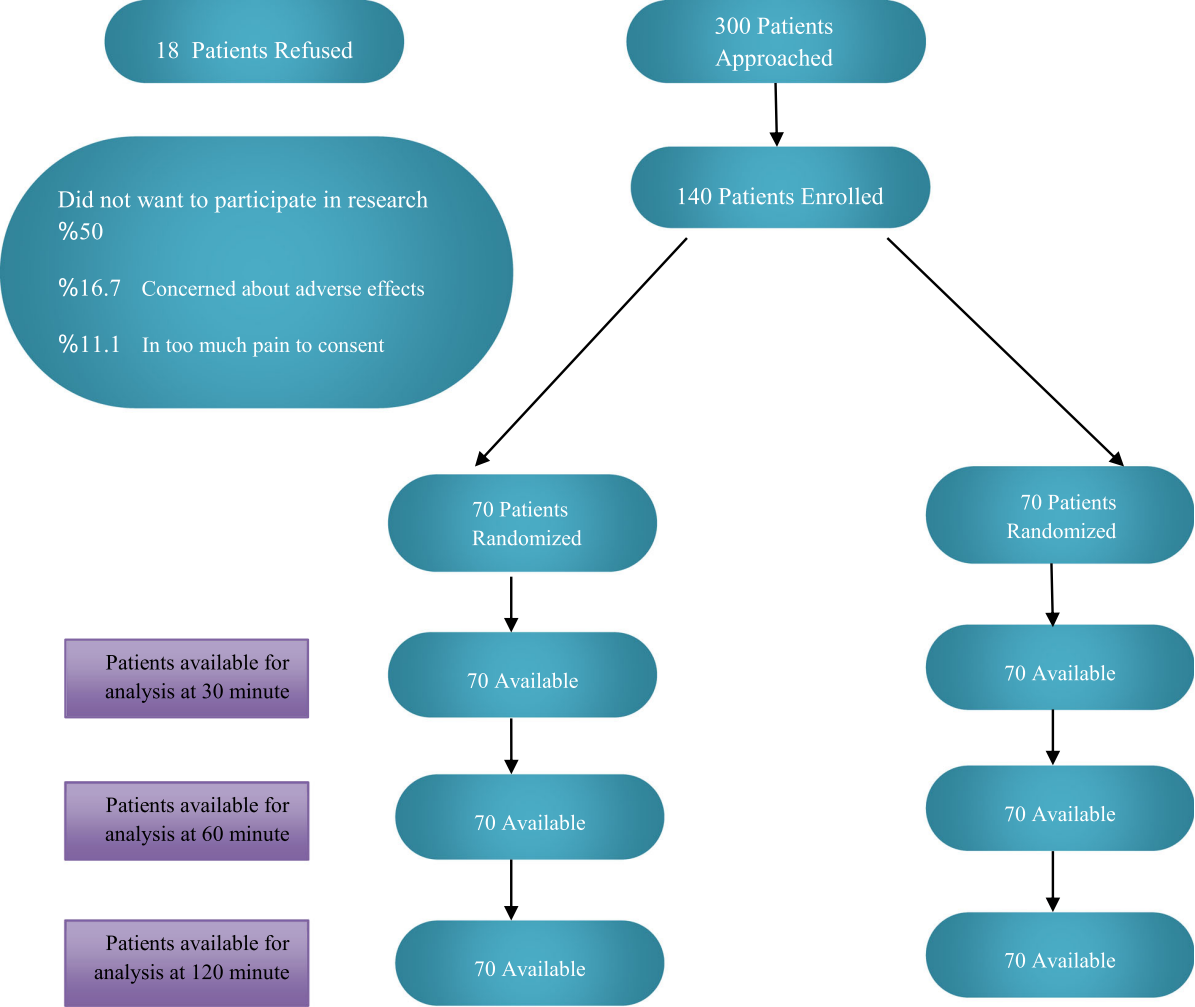
CONSORT flow diagram of the study

Three hundred people expressed willingness to participate in the study, 102 of whom were excluded from the study due to failing to meet inclusion data. Eighteen patients refused to participate in the study, and finally, out of 140 eligible patients, 70 patients were randomly assigned to the intranasal ketamine group and another 70 to the intravenous ketorolac group (Fig. 1). The mean age of the study patients was 41.6 ± 16.6 years. The age, sex, medical history, history of drug sensitivity, and history of the two groups’ headache did not show significant differences (Table 1).

**Table 1:** Patient Baseline Characteristics

Characteristic	Ketamine	Ketorolac	*P* value
Sex			0.865
male	32 (45.7)	33 (47.1)	
female	38 (55.3)	37 (52.9)	
Age			0.594
< 30	23 (32.9)	23 (32.9)	
31–40	10 (14.3)	18 (25.7)	
41–60	11 (15.7)	14 (20)	
> 60	26 (37.1)	15 (21.4)	
Drug history			0.080
yes	11 (16)	24 (34)	
no	59 (84)	46 (64)	
Sensitivity drug history			0.080
yes	0 (0)	3 (4)	
no	70 (100)	67 (96)	
Headache history			0.716
yes	47 (67)	49 (70)	
no	23 (33)	21 (30)	

Values are presented as number (%)

The mean pain intensity of the two groups at all time points was significantly different, according to Repeated-measures ANOVA (*P* < 0.001). According to this test, the ketamine group’s pain intensity was lower than the ketorolac group at all time points except the 120th minute. The mean reduction of pain intensity in the first 30 min in the ketamine group (4.53 ± 1.25) was higher than the ketorolac group (4.03 ± 0.98), which was a significant difference (*p* = 0.003). The greatest decrease in pain intensity was observed in the first 60 min in the ketorolac group (6.58 ± 1.03), which was also significantly different (*p* = 0.005). The mean reduction of pain intensity in the ketorolac and ketamine group after 120 min was (8.01 ± 0.69) and (6.90 ± 1.20), respectively, significant differences were detected between the two groups (*p* < 0.001). Between 60 and 120 min, the mean reduction of pain severity in the ketorolac group (0.69 ± 1.43) was greater than the ketamine group (0.42 ± 0.97), which was a significant difference (Table 2).

**Table 2:** Pain Score of Subjects at Different Time Intervals Based on Visual Analogue Scale

Time	Ketamine (*n* = 70)		Ketorolac (*n* = 70)		
	Mean ± SD	Median (IQR)	Mean ± SD	Median (IQR)	P value
On arrival	8.00 ± 1.1	8.0 (2.0)	9.01 ± 0.7	9.0 (3.0)	< 0.001
30	3.47 ± 0.5	3.0 (1.0)	4.98 ± 0.7	5.0 (0.0)	< 0.001
60	2.07 ± 0.2	2.0 (0.0)	2.42 ± 0.7	2.0 (1.0)	< 0.001
120	1.10 ± 0.3	1.0 (0.0)	1.00 ± 0.0	0.0 (0.0)	< 0.001
Pain reduction until 30th minute	4.53 ± 1.25	4.5 (4,5)	4.03 ± 0.98	4 (3,5)	0.003
Pain reduction until 60th minute	5.93 ± 1.15	6 (5,7)	6.58 ± 1.03	7 (6,7)	0.005
Pain reduction until 120th minute	6.9 ± 1.22	7 (6,7)	8.01 ± 0.69	8 (8,8)	< 0.001
Pain reduction 60 to 120th minute	0.97 ± 0.42	1 (1,1)	1.43 ± 0.69	1 (1,2)	< 0.0001

SD = standard deviation; IQR = interquartile

**Fig. 2:**
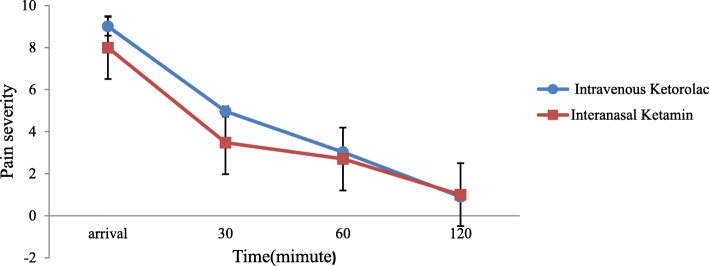
Changes of pain severity based on VAS in patients receiving intravenous ketorolac and intranasal ketamine versus during the study period. VAS: Visual Analogue Scale

A two-way comparison of the mean pain intensity in patients in both groups at different time points showed that the pain intensity in both groups decreased within 120 min, which was statistically significant (*p* < 0.001) (Fig. 2).

### Side effects

No subjects were excluded from the study due to side effects. Dizziness, nausea, increased heart rate, and increased blood pressure were statistically significant differences between the ketamine and ketorolac groups (*p* < 0.05). Among all the side effects, the most and the least side effects were related to increased heart rate and general discomfort, respectively. Generally, side effects were greater in the ketamine group. Details of side effects have been reported in Table 3.

**Table 3:** Frequency of side effects in the study population

Side effect	Ketamine (*n* = 70)	Ketorolac (*n* = 70)	P value
Fatigue	5 (7.14)	2 (2.86)	0.245
Dizziness	11 (15.71)	2 (2.86)	0.009
Discomfort (generalized)	2 (2.86)	0	0.154
Nausea	8 (11.43)	2 (2.86)	0.049
HR rising	28 (40.00)	11 (15.71)	0.001
Blood pressure rising	9 (12.86)	23 (32.86)	0.005
*Other*	2 (2.86)	4 (5.71)	0.404

Values are presented as number (%)

## Discussion

The present study was conducted to compare the efficacy of intranasal ketamine and intravenous ketorolac on acute non-traumatic headaches in patients referred to the emergency department. The results showed that intranasal ketamine and intravenous ketorolac effectively and almost similarly reduced patients’ headaches, while ketamine in the short term and ketorolac in the long term further reduced the severity of pain. Also, side effects such as fatigue, dizziness, general malaise, nausea, and increased heart rate caused by ketamine were far greater than ketorolac. Non-traumatic headaches are one of the most common complaints of patients referred to emergency departments [29]. NSAIDs, neuroleptics, magnesium sulfate, and triptan are used to manage and treat headaches [30]. Ketamine is used as a sedative drug to manage and treat headaches [15, 17, 31]. Sadove et al. were among the first to study the effect of low-dose ketamine [32]. However, the effectiveness of ketamine as an analgesic is still being discussed. Various studies have shown the antihyperalgesic effect of ketamine is stronger than its analgesic properties [33, 34]. Few studies have examined the effect of intranasal ketamine on the severity of headaches. The present study results showed that in the first 30 min, ketamine intranasal reduced the severity of pain more than intravenous ketorolac. Andolfatto et al., in their study on the effectiveness of intranasal ketamine for analgesia in the emergency department patients, found that intranasal ketamine reduced the VAS pain score in 88% of patients in emergency department and relieved the pain quickly and effectively [35]. The effect of ketamine intranasal on treating patients with severe pain in the emergency department was investigated by Shrestha et al. (2016). The results indicated a significant reduction in the severity of pain in patients [36]. Additionally, Benish in a study compared analgesia with metoclopramide and diphenhydramine versus intranasal ketamine on patients with primary headache. The results showed that VAS changes were greater after 30 min in the ketamine group (29.0 mm) compared to the metoclopramide-diphenhydramine group (22.2 mm) [20]. One of the most important advantages of ketamine is its various prescription methods [20, 37]. Ketamine acts through various mechanisms, including the supraspinal mechanisms, cholinergic and monoamine effects, local anesthetic action, sigma receptor interaction, and NMDA receptor antagonism [34]. These mechanisms can be accompanied by favorable or unfavorable clinical effects [38]. Among the favorable effects can be noted to the lack of inducing platelet function disorders and the suitable replacement for morphine in patients with asthma (due to histamine release in response to morphine) [34, 39]. Adverse effects of ketamine may manifest in various forms, including increased systolic blood pressure, tachycardia, nausea, vomiting, fatigue, dizziness, discomfort, mood change, and feeling of unreality [17, 19, 28, 40]. In the present study, the incidence of side effects, including fatigue, dizziness, general discomfort, nausea, and increased heart rate, was higher in the ketamine group. Also, due to the irritability and low threshold of conscious impairment following ketamine administration, physicians are not willing to prescribe this drug [38, 41]. Ketorolac is an NSAID with analgesic and anti-inflammatory properties [42]. According to a systematic review by Taggart et al., intravenous ketorolac has been recommended as a second-line drug in the management of migraine [26]. In this regard, the results of other studies have shown that ketorolac may take effect after 30 to 60 min [43, 44]. Baratloo et al. in their study examined the efficacy measurement of ketorolac in reducing the severity of the headache. The results showed that ketorolac reduced the severity of pain in the first 60 min more than in the second 60 min [10]. The results of a study by John et al., conducted to compare the effectiveness of nasal sumatriptan versus intravenous ketorolac on migraine headaches, showed that intravenous ketorolac reduced pain more than sumatriptan after one hour [18]. Our results are in line with this study. Additionally, Kasmaei et al. (2017) concluded that magnesium sulfate and ketorolac effectively reduced pain in patients with acute migraine headaches, but the reduction in pain by magnesium sulfate in the first and second hours after administration was greater than ketorolac [30]. The difference in results between our study and this study was probably due to different methods and sample sizes.

The strengths of our study are double-blind and a large sample size. The doubling of the sample size compared to the required sample size for the present study indicates the study’s strength and the generalizability of the data to the general population.

### Study limitations

The present study’s first limitation was the diversity of patients ‘mental responses to the mental perception of pain and their encouragement to participate in the study. Patients’ perception of pain is affected by drug pharmacokinetics and is sometimes different. Another limitation was that interventions made on all patients were affected, and the ineffectiveness of the interventions was not observed in any of the subjects. Another important limitation of this study was that patients entered the study when the investigators were available. The times of the investigator’s presence in the hospital included morning, evening shifts on all days of the week; however, headache patients presenting to ED were not recorded during times when investigators were not available. Also, the date and time of referral of these patients, as well as the date and time of the investigator’s absence, were not regularly recorded. According to the results of the present study, although intranasal ketamine further reduced pain in a short time, it had more side effects than ketorolac, it is recommended that future studies should be investigated the effectiveness of these two drugs on different categories of headaches, the long-term effects of ketamine and ketorolac as well as other side effects of these two drugs.

## Conclusion

The present study showed that intranasal ketamine had more analgesic effect than intravenous ketorolac in a shorter time, and it can be used as a selective drug for the management and treatment of headaches.

## Data Availability

Data will be available by contacting the corresponding author.

## Abbreviations

ED: Emergency department
KUMS: Kermanshah University of Medical Sciences
